# Randomized trial studying metabolic outcomes and quality of life after adrenalectomy versus conservative management for mild autonomous cortisol secretion

**DOI:** 10.1530/EC-25-0361

**Published:** 2025-07-19

**Authors:** Grethe Å Ueland, Oskar Ragnarsson, Anette Heie, Albin Kjellbom, Ola Lindgren, Andreas Muth, Fausto Palazzo, Per L Poulsen, Lars Rolighed, Hrafnkell Baldur Thordarson, Florian Wernig, Anders Bergenfelz

**Affiliations:** ^1^ Department of Medicine, Haukeland University Hospital, Bergen, Norway; ^2^ Department of Endocrinology, Sahlgrenska University Hospital, Gothenburg, Sweden; ^3^ Department of Internal Medicine & Clinical Nutrition, Institute of Medicine, Sahlgrenska Academy, University of Gothenburg, Gothenburg, Sweden; ^4^ Departments of Breast and Endocrine Surgery, Haukeland University Hospital, Bergen, Norway; ^5^ Lund University, Faculty of Medicine, Department of Clinical Sciences Lund, Lund, Sweden; ^6^ Department of Endocrinology, Skåne University Hospital, Lund, Sweden; ^7^ Department of Surgery, Institute of Clinical Sciences, Sahlgrenska Academy, University of Gothenburg, Gothenburg, Sweden; ^8^ Department of Surgery, Sahlgrenska University Hospital, Region Västra Götaland, Gothenburg, Sweden; ^9^ Hammersmith Hospital, Imperial College Healthcare NHS Trust, London, UK; ^10^ Steno Diabetes Center Aarhus (SDCA), Aarhus University Hospital, Aarhus, Denmark; ^11^ Department of Surgery and Department of Otorhinolaryngology, Aarhus University Hospital, Aarhus, Denmark

**Keywords:** adrenal incidentalomas, mild autonomous cortisol secretion, MACS, adrenalectomy, metabolic consequences

## Abstract

**Objective:**

Evaluate the impact of adrenalectomy on metabolic parameters and quality of life (QoL) in patients with mild autonomous cortisol secretion (MACS).

**Method:**

A multicenter prospective randomized clinical trial compared adrenalectomy with conservative management. Metabolic parameters and QoL were assessed at baseline and after 2 years.

**Results:**

Forty-three MACS patients with a single adrenal adenoma were randomized to either adrenalectomy (*n* = 21) or conservative management (*n* = 22). At baseline, 33 patients had hypertension, 13 had type 2 diabetes (T2D), 18 used statins, and nine patients had osteoporosis. After 2 years, normalization of cortisol levels post 1 mg dexamethasone suppression test was achieved in 19/21 adrenalectomy patients compared to 2/22 patients in the conservative group (*P* < 0.01). All adrenalectomy patients had a significant increase in ACTH and DHEA-S. Office blood pressure and daily defined doses of antihypertensives (DDD) improved in nine of 12 adrenalectomy patients versus four of 15 conservatively treated patients (*P* = 0.01). Using 24 h blood pressure and DDD, improvement rates were five of 11 in the adrenalectomy group and six of 15 in the conservative group (*P* = 0.78). Among patients without T2D, the 120 min glucose level during oral glucose tolerance test was lower in the adrenalectomy group (6.2 vs 7.3 mmol/L, *P* = 0.04), but within-group changes were not different (*P* = 0.76). There were no statistically significant differences in QoL between the two groups.

**Conclusion:**

Adrenalectomy showed trends toward improvement in office blood pressure and glucose metabolism in MACS, suggesting possible reduction in cardiovascular risk and metabolic complications.

**Clinical trials number:**

NCT01246739

## Introduction

Cortisol is an important regulator of stress and metabolism, and its secretion shows distinct circadian and ultradian rhythms. Excess cortisol, as in patients with Cushing syndrome (CS), is associated with high morbidity and mortality (1, 2). In recent decades, it has become evident that even mild overproduction of cortisol, termed ‘mild autonomous cortisol secretion’ (MACS), may have adverse metabolic and psychological effects (3, 4).

MACS is associated with the metabolic syndrome, and studies have shown increased prevalence of type 2 diabetes (T2D) and hypertension (5, 6, 7, 8). Obesity and hypercholesterolemia are overrepresented in this patient group (5, 9). In addition, MACS negatively affects bone health, with increased prevalence of osteoporosis and osteoporotic fractures (10). The negative health effects of MACS contribute to higher cardiovascular event rates and increased mortality (6, 11), particularly in women (3). The role of adrenalectomy in reducing cardiovascular risk factors and improving bone health in patients with MACS remains debated. While some studies indicate improvements in metabolic parameters following adrenalectomy (12, 13, 14, 15, 16), others suggest minimal benefit (17, 18). Lacobone *et al.* demonstrated in 2012 significant improvements in SF-36 quality of life scores in patients undergoing adrenalectomy compared to conservative management (13); however, to date, no randomized controlled trials on quality of life have been conducted in this field. The need for comprehensive, randomized, prospective studies with long-term follow-up focusing on cardiovascular endpoints and mortality data is highlighted in the latest European adrenal incidentaloma guidelines (19). Current guidelines for treatment of MACS advocate an individualized approach to MACS management, considering metabolic disease burden and patient preference (1).

This multicenter randomized controlled trial investigated the hypothesis that adrenalectomy would yield superior outcomes compared to conservative management in patients with MACS. Specifically, we studied any improvements in blood pressure control, glucose metabolism, body mass index (BMI), and quality of life following surgical intervention. In this report, we present the 2-year follow-up data for both the surgical and conservative management groups, which offers insights into the long-term efficacy of these contrasting approaches.

## Method

### Trial design

From September 2012 to December 2023, we consecutively included patients with unilateral adrenal incidentalomas diagnosed with MACS at five large hospitals in Sweden, Norway, Denmark, and the UK. Patients were randomized to adrenalectomy (intervention) or conservative follow-up (control).

Computer-based randomization in blocks of six was performed centrally (Lund), and prepared envelopes were sent to each center, which carried out randomization locally by drawing envelopes. The study was unblinded.

### Participants

Patients evaluated for adrenal incidentalomas at the outpatient clinics of participating centers and diagnosed with MACS were eligible for inclusion. MACS was defined as a morning cortisol level above 50 nmol/L following a 1 mg overnight dexamethasone suppression test (DST), and a low morning basal plasma ACTH (<2.2 pmol/L or <7 ng/L). In addition, 24 h urine free cortisol (UFC) had to be within normal limits to exclude overt CS.

### Interventions

All patients underwent office blood pressure measurement (measured three times with the average of the last two measurements), 24 h blood pressure monitoring, cardiac ultrasound, bone density measurement, and completed the SF-36 questionnaire at baseline. Weight and height were measured in the office. In non-diabetic patients, a 75 gram oral glucose tolerance test (OGTT) was performed, with measurement of fasting glucose before the test and glucose at 120 min after the test. The same procedures were performed at the 2-year follow-up (defined as a visit 2 years plus/minus 3 months from the time of inclusion). The procedures were not standardized across centers.

Patients in the intervention group underwent minimally invasive adrenalectomy. The control group was followed with conservative management. This involved optimization of the treatment of metabolic complications at baseline and no standardized follow-up until the 2-year follow-up. Demographic data, clinical and biochemical data were collected along with validated quality of life measurements (SF-36).

The analyses were conducted per protocol.

### Definitions

Postoperative biochemical cure was defined as a post-DST cortisol level below 50 nmol/L, and ACTH in the normal reference range.

Hypertension was defined as an office blood pressure measured above 140/90 mmHg at baseline in a patient without known hypertension or an already established diagnosis of hypertension, with or without antihypertensive drugs.

Daily defined doses of antihypertensives (DDD) were calculated using a calculator from the National Institute of Public Health (20).

Decreased office blood pressure was defined as a reduction in systolic and/or diastolic blood pressure of 10 mmHg or more. For 24 h blood pressure monitoring, a reduction of 10 mmHg in systolic and/or 5 mmHg in diastolic average blood pressure was considered reduced blood pressure. Conversely, increased blood pressure was defined similarly but in the opposite direction.

In this study, an improvement of blood pressure was defined according to the following three criteria: i) normalization of hypertension in patients not on medical treatment, ii) unchanged or decreased blood pressure in a patient with hypertension with reduction in DDD of antihypertensives by 0.5 or more, or iii) decreased blood pressure in patients with hypertension and unchanged DDD of antihypertensives.

Worsening of blood pressure was defined according to the following criteria: i) development of office blood pressure above 140/90 mmHg in a patient without known hypertension at baseline, ii) unchanged or increased blood pressure in a patient with hypertension with increase in DDD of antihypertensives by 0.5 or more, or iii) increased blood pressure in patients with hypertension and unchanged DDD of antihypertensives.

Diabetes was defined as an established diagnosis of T2D, or plasma HbA1c equal to or above 48 mmol/moL, or a two-hour blood glucose level of 11.1 mmol/L or more following an OGTT.

Improvement or worsening of glucose metabolism was defined as a change in HbA1c of more than 2 mmol/moL, or decreased/increased medication for T2D.

Left ventricular hypertrophy was defined as a septal thickness of 10 mm or more.

Postoperative adrenal insufficiency was defined as a serum morning cortisol postoperatively below 300 nmol/L or a 60 min cortisol level below 500 nmol/L after a 250 microgram Synacthen test.

### Hormone assays

All hormone analyses were performed at local laboratories using validated assays (details in Supplemental material (see section on Supplementary materials given at the end of the article)).

### Quality-of-life measurement

The Short Form Health Study (SF-36) was used to evaluate quality of life. The form is generic and maps physical and mental symptoms, impaired function, pain, vitality, and well-being. The Norwegian, Swedish, Danish, and English versions are all validated and have demonstrated good cross-cultural validity, with scores ranging from 0 to 100 (higher scores indicating better health).

### Statistical analyses

Data were analyzed using the Statistical Package for the Social Sciences (SPSS Version 29.0; IBM Corporation, USA). Median, range, and percent were used to describe the cohorts. Mann–Whitney U test and RR with 95% confidence interval (CI) were used to describe differences between groups as appropriate. A two-tailed *P*-value <0.05 was considered statistically significant.

An initial power calculation indicated that, with 80% power and the significance level set at 95%, 27 patients in each arm would be required to address the primary study endpoint: improvement of hypertension according to the study definition.

### Ethics

The study was approved by local ethics committees for each center. All participants provided written informed consent. The research was conducted according to the Declaration of Helsinki. Clinical trials number: NCT01246739.

## Results

### Subject characteristics

**Figure 1:**
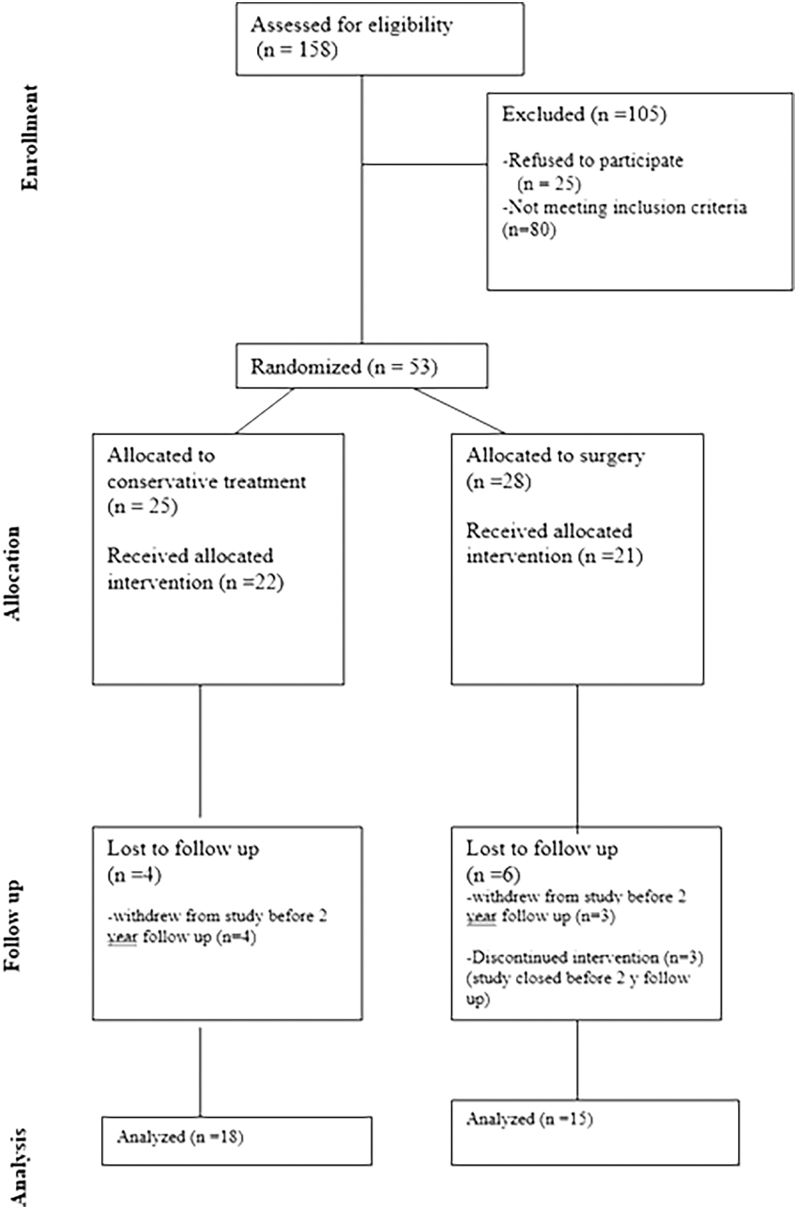
CONSORT diagram showing the flow of participants through each stage of the trial.

Fifty-three patients were randomized, whereas forty-three received the allocated intervention: 22 to conservative follow-up, and 21 to adrenalectomy. Figure 1 shows a CONSORT diagram of the flow of participants through each stage of the trial. Table 1 displays the baseline clinical characteristics of both patient groups. Baseline characteristics, including age, sex, BMI, and smoking status, were similar between the groups, and baseline biochemical profiles were comparable.

**Table 1:** Showing baseline characteristics of the conservative and surgery group, respectively.

	Conservative group	Surgery group	*P*-value
	*n* = 22	*n* = 21	
Women, *n* (%)	15 (68)	11 (52)	0.30
Age, years median (range)	65 (55–80)	63 (35–73)	0.26
BMI kg/m^2^	29.6 (21.2–42.2)	28.0 (21.7–36.0)	0.46
Obese, *n* (%)	9 (41)	7 (33)	0.62
Smoking, *n*	6 (27)	9 (43)	0.26
Biochemistry			
Creatinine, *μ*mol/L	71 (56–156)	73 (48–229)	0.70
s-cortisol morning, nmol/L	517 (240–697)	425 (154–636)	0.39
p-ACTH morning, pmol/L	1.5 (0.2–3.8)	1.4 (0.2–1.9)	0.43
UFC, nmoL/24 h	44 (19–264)	64 (2–147)	0.42
DHEA-S, *μ*mol/L	0.6 (0.17–2.6)	1.2 (0.4–4.2)	0.21
Cortisol after DST, nmol/L	94 (64–381)	101 (55–402)	0.91
s-glucose baseline, mmol/L	6.0 (3.6–7.1)	5.8 (4.4–8.1)	0.71
s-glucose 120 min, mmol/L*^,^^†^	7.5 (5.3–13.2)	7.6 (3.4–15.2)	1.0
HbA1C mmol/moL	39 (32–58)	39 (32–56)	0.85
Metabolic comorbidities			
Hypertension, *n*	18 (82)	17 (81)	0.93
Diabetes mellitus, *n*	6 (27)	7 (33)	0.67
Osteoporosis, *n*	5 (23)	4 (19)	0.65
Fractures, *n*	7	4	0.34
Supplemental tests			
Office systolic BP, mmHg	138 (124–186)	135 (111–180)	0.74
Office diastolic BP, mmHg	81 (58–112)	80 (68–97)	0.76
Mean 24 h systolic BP, mmHg^†^	140 (105–185)	131 (87–188)	0.33
Mean 24 h diastolic BP, mmHg^†^	82 (57–95)	79 (59–104)	0.76
Medications, *n* (%)			
Daily doses antihypertensive	1.8 (0–5)	1.4 (0–6.5)	0.94
Cholesterol lowering drugs, *n*	10 (45)	8 (38)	0.63
Tumor characteristics			
Tumor size, mm	35 (22–40)	28 (10–51)	0.07
Tumor density, HU	2 (−17–20)	5 (−15–39)	0.43

Continuous variables are presented as median (range), and categorical variables are given as number (*n*) and percent (%). BMI, body mass index; DST, dexamethasone suppression test; UFC, 24 h urine free cortisol; BP, blood pressure; HU, Hounsfield units; DHEA-S, dehydroepiandrostendione sulfate. Obese means BMI ≥30 kg/m^2^.

*P*-values indicate differences between the conservative and surgery groups at baseline.

Reference ranges for s-cortisol morning, p-ACTH, and UFC are given in the supplemental file, as they are slightly different between the hospitals. Reference ranges for DHEA-S (<2.5 μmol/L), cortisol after DST (<50 nmol/L), s-fasting glucose (<7 mmol/L), and s-glucose 120 min post oral glucose tolerance test (≤11.1 mmol/L).

* In non-diabetic patients (*n* = 30).

^†^ At baseline, the number of patients with missing data in the conservative and surgery groups, respectively, was as follows: 24 h BP (0/1), OGTT in non-diabetic patients (2/0), septum thickness (3/5), and bone density measurement (4/3).

A significant proportion of patients had hypertension at baseline (18/22 in the conservative group, and 17/21 in the adrenalectomy group). Type 2 diabetes (T2D) was also prevalent, affecting 6/22 patients in the conservative group, and 7/21 in the adrenalectomy group at baseline. Moreover, 18/43 patients were on statin therapy at inclusion (ten in the conservative group, eight in the surgery group), and approximately one-fifth of all included patients with MACS had established osteoporosis upon enrollment (five in the conservative group, and four in the surgery group).

### Hypercortisolism

Cortisol normalized in 19/21 patients who underwent adrenalectomy versus 2/22 in the conservative group at 2 years (Table 2). Both non-normalized surgical patients achieved clinical cure. The first patient showed improvement in hypertension and weight loss, with ACTH levels increasing from suppressed to the mid-normal range. In addition, the patient required glucocorticoid replacement therapy for 3 months following surgery, indicating transient adrenal insufficiency. The second patient also achieved normalization of ACTH levels, but started oral estrogen replacement following the operation, which is known to increase the cortisol-binding globulin concentration, potentially giving a false-positive post-DST cortisol result. Five of the operated patients experienced postoperative adrenal insufficiency, lasting from 3 to 6 months.

**Table 2:** Showing 2-year follow-up data of the conservative and surgery groups, respectively.

	Conservative group	Surgery group	*P*-value
	*n* = 18	*n* = 15	
Women, *n* (%)	13 (72)	8 (53)	
Age, years median (range)	68 (59–82)	65 (37–75)	
BMI kg/m^2^	31.2 (21.1–43.1)	28.6 (22.3–34.7)	0.44
Obese *n* (%)	9 (50)	6 (40)	0.53
Δ BMI kg/m^2^	0 (−2.9–4.9)	−0.2 (−2.2–8.4)	0.94
Biochemistry			
p-ACTH morning, pmol/L	1.8 (1.1–3.2)	6.5 (3.5–11.1)	<0.01
UFC (24 h urine free cortisol), nmoL/24 h	32 (17–76)	47 (43–117)	0.35
DHEA-S, *μ*mol/L	0.47 (0–2.0)	2.5 (1–4.0)	0.06
Cortisol after DST, nmol/L	95 (33–231)	43 (10–98)	<0.01
s-glucose baseline, mmol/L	5.6 (4.9–8.6)	5.2 (4.4–6.9)	0.98
Δ s-glucose baseline, mmol/L	0.5 (−2.4–2.6)	0.05 (−2.2–3.5)	0.72
s-glucose 120 min, mmol/L*^,^^†^	7.3 (4.8–18.1)	6.2 (3.8–6.8)	0.04
Δ s-glucose 120 min, mmol/L	−0.6 (−4.8–8)	−0.65 (−1.8–0.4)	0.76
Blood pressure			
Office systolic BP, mmHg^†^	138 (124–157)	125 (106–165)	0.03
Office diastolic BP, mmHg^†^	81 (70–93)	75 (55–115)	0.27
Δ office systolic BP, mmHg	1 (−40–17)	−13 (−38–47)	0.08
Δ office diastolic BP, mmHg	1 (−40–17)	−6 (−23–28)	0.17
Mean 24 h systolic BP, mmHg^†^	130 (106–193)	122 (107–164)	0.32
Mean 24 h diastolic BP, mmHg^†^	78 (63–109)	75 (62–93)	0.38
Δ Mean 24 h systolic BP, mmHg	−7 (−37–63)	−8 (−57–49)	0.92
Δ Mean 24 h diastolic BP, mmHg	−4 (−15–25)	−6 (−31–25)	0.57
Medications, *n* (%)			
Daily doses antihypertensive^†^	2.0 (0–8.5)	2.3 (0–6.5)	0.65
Δ Daily doses antihypertensive	0 (−3–3.5)	0 (−1–2.5)	0.17

Continuous variables are presented as median (range), and categorical variables are given as number (*n*) and percent (%). BMI, body mass index; DST, dexamethasone suppression test; UFC, 24 h urine free cortisol; BP, blood pressure; HU, Hounsfield units; DHEA-S, dehydroepiandrostendione sulfate. Obese means BMI ≥30 kg/m^2^.

*P*-values indicate differences between the conservative and surgery groups at baseline.

Reference ranges for s-cortisol morning, p-ACTH, and UFC are given in the supplemental file, as they are slightly different between the hospitals. Reference ranges for DHEA-S (<2.5 μmol/L), cortisol after DST (<50 nmol/L), s-fasting glucose (<7 mmol/L), and s-glucose 120 min post oral glucose tolerance test (≤11.1 mmol/L).

Δ illustrates the median (range) change in the parameter from baseline to 2-year follow-up in the two groups.

* In non-diabetic patients (*n* = 30).

^†^ At the 2-year follow-up, the following numbers of patients had missing data in the conservative and surgery groups, respectively: office BP (3/8), DDD (2/3), 24 h BP (3/7), OGTT (4/7).

### Blood pressure/cardiac hypertrophy

Office systolic blood pressure was lower at 2-year follow-up in patients treated with adrenalectomy compared to conservatively treated patients (125 vs 138 mmHg, *P* = 0.03, Table 2). However, when accounting for baseline systolic blood pressure, the change in blood pressure between the groups (Δ systolic blood pressure) was not significant (*P* = 0.08). The office diastolic blood pressure at 2 years was not different between the groups. Furthermore, the 24 h systolic and diastolic blood pressure at 2 years was not different between the groups.

Among conservatively treated patients with hypertension, nine maintained an unchanged dose of antihypertensive medication, six had an increased DDD, and one patient had a reduced DDD. None of the four patients without hypertension at baseline had developed it at 2 years. Among patients with hypertension at baseline who were treated with adrenalectomy, seven patients maintained an unchanged dose, two had increased DDD, and five experienced a reduction.

**Figure 2:**
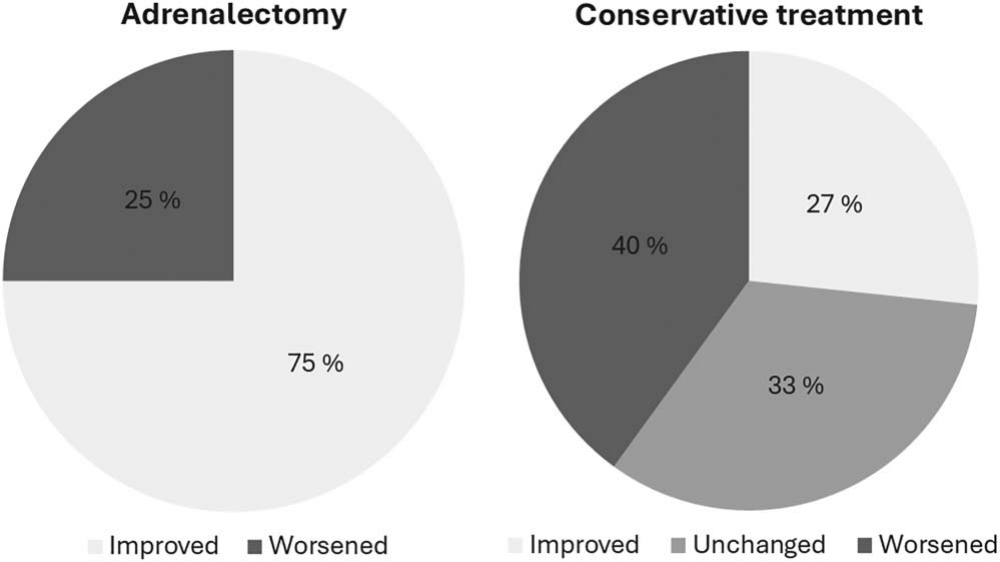
Showing the percentage of patients from each group that had improved, unchanged, or worsened blood pressure from baseline to the 2-year follow-up (based on evaluation of changes in office blood pressure and DDD).

At 2 years, by evaluating changes in office blood pressure and DDD, nine of 12 patients with hypertension in the adrenalectomy group had improved blood pressure compared to four of 15 patients who received conservative treatment (*P* = 0.01), as shown in Fig. 2. However, by using changes in 24 h blood pressure and DDD, a similar number of patients treated with adrenalectomy (five of 11) and conservatively treated patients (six of 15) had improved blood pressure at 2 years (*P* = 0.78).

Septum thickness at baseline was available for 19 conservative and 16 surgery patients, with six in each group having left ventricular hypertrophy. At the 2-year follow-up, two more conservative patients developed left ventricular hypertrophy, and none in the surgery group.

### Glucose metabolism

At 2 years, glycemic outcomes among T2D patients were similar in both groups: in the conservative group (*n* = 5), two worsened, one improved with more medication, and one was unchanged; in the surgery group (*n* = 5), two worsened, two were unchanged, and one improved.

Among patients without T2D, HbA1c was unchanged, improved, or worsened in equal proportions in both groups. New-onset T2D developed in two conservative and one surgical patient.

At follow-up, 120 min OGTT glucose was significantly lower in the surgery group (*P* = 0.04), but the change from baseline (Δ 120 min glucose) was not significantly different between groups (*P* = 0.16).

### BMI/weight

In the conservative group, there was no significant median weight change at the 2-year follow-up (range −9 to +14) kg. In the surgery group, patients experienced a median weight loss of −1.5 kg (range −16 to +25), although the difference was not statistically significant (*P* = 0.47). Among the 19 conservatively managed patients with baseline and 2-year follow-up data available on weight/BMI, six patients had unchanged weight, six patients lost weight, and seven patients gained weight. In the surgery group, with 16 patients providing comparable data, four patients had unchanged weight, eight patients lost weight, and four patients gained weight. At the group level, the conservative group showed an increase in BMI from a median of 29.6 kg/m^2^ at baseline to 31.2 kg/m^2^ at follow-up. In contrast, the surgery group maintained a stable BMI over the same period (*P* = 0.44).

### Bone metabolism

There were no differences between the groups in total bone mass or *t*-score at any measured location (back and hip).

### Quality-of-life (SF-36)

**Figure 3:**
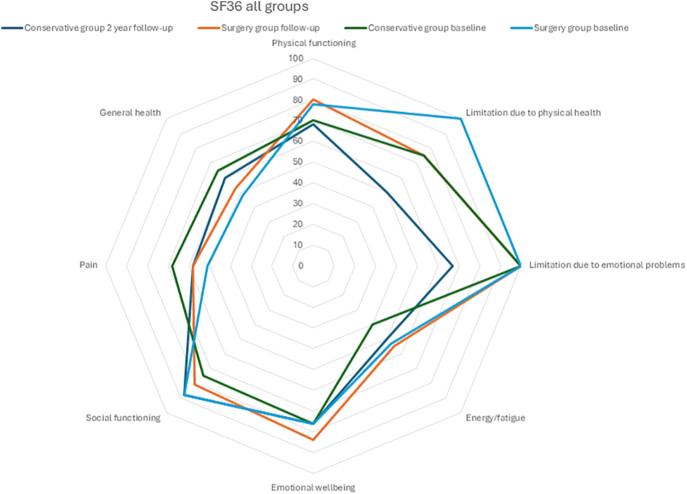
Radar plot showing the quality of life data (SF-36) at baseline and 2-year follow-up in the conservative group and the surgery group.

There were no statistically significant differences in quality-of-life scores between the two groups, as measured by the SF-36 questionnaire (Fig. 3 and Supplemental Table 3).

## Discussion

This international multicenter randomized clinical trial (RCT) compared adrenalectomy with conservative management in patients with MACS and demonstrated that a higher proportion of patients in the adrenalectomy group achieved improved blood pressure compared to those managed conservatively. In addition, among patients without T2D, the adrenalectomy group showed significantly lower 120 min OGTT glucose at follow-up, but change from baseline did not differ. Since baseline values were similar, this may reflect small sample size and limited power to detect differences in change scores.

A 2024 systematic review and meta-analysis by Khadembashiri *et al.* compared adrenalectomy with conservative treatment in patients with MACS (21). The study found that adrenalectomy was associated with greater improvements in metabolic outcomes, cardiovascular risk factors, and quality of life. However, the authors noted significant heterogeneity among studies and emphasized the need for more high-quality randomized trials.

In a guideline published in 2023 (19), the expert group conducted a review of the existing literature on treatment of MACS and its impact on metabolic parameters (22). This review identified nine observational studies (12, 13, 15, 23, 24, 25, 26, 27, 28) and only two RCTs (14, 29). The two RCTs had small sample sizes and lacked standardization of endpoints. Subsequently, a third RCT addressed these issues, including a larger sample size and standardized outcomes (30). However, further studies are needed to more thoroughly assess the metabolic and cardiovascular benefits of adrenalectomy in MACS, to include more diverse patient populations, and to provide longer-term follow-up. Our study evaluated endpoints in a standardized manner and recorded antihypertensive medication use as DDD, facilitating accurate comparison and interpretation of therapeutic effects.

In 2009, Toniato *et al.* conducted the first RCT on this topic, involving 45 patients with a mean follow-up period of 7.7 years (14). While improvements in blood pressure, glucose and lipid metabolism, and weight were observed in the adrenalectomy group, the endpoints were not standardized, and OGTT was not performed in non-diabetic patients. Morelli *et al.* conducted a subsequent RCT with 62 patients (55 completed the trial), but limited follow-up to 6 months (29). This study showed improvement in glucose metabolism and blood pressure in the surgery group. Although glucose data, including HOMA index, were robust, the study did not calculate DDD for antihypertensive medications. Quality-of-life outcomes were not assessed.

The most comprehensive RCT to date was conducted by Koh *et al.* and published in Annals of Surgery in 2024 (30). The study included 132 patients with a 48-month follow-up and demonstrated improvements in blood pressure and glucose metabolism in the adrenalectomy group.

The most recent randomized controlled trial on the topic was published in May 2025. The study included 52 patients and concluded that minimally invasive adrenalectomy can safely improve secondary hypertension associated with MACS in unilateral adrenal incidentalomas (16).

Regarding quality of life, Iacobone *et al.* demonstrated significant improvements in SF-36 scores in patients undergoing adrenalectomy compared to conservative management (13). Our study similarly observed improvements across all eight SF-36 domains in the adrenalectomy group, although these findings did not reach statistical significance, most probably due to the small sample size. Quality of life remains a valuable outcome for future research, as it could serve as an additional criterion in treatment decisions for patients with MACS.

The primary limitation of our study was the small size of the cohort. The pre-study power analysis indicated a requirement of 54 patients (27 per group) to detect a significant difference in the primary outcome (improvement in blood pressure). However, slow recruitment and, importantly, challenges for follow-up affected by the COVID-19 pandemic resulted in the enrollment of only 43 patients. A post hoc power analysis, based on the observed effect size, revealed that 80 patients (40 per group) would have been needed to detect a statistically significant difference at the five percent level and with 80 percent power. In addition, the procedures for measurement of blood pressure and ultrasound of the heart were not standardized, and there was no predefined protocol for escalation/de-escalation of antihypertensive and glucose-lowering agents. For bone density and cardiac hypertrophy, there were substantial missing data, making it impossible to discuss or draw conclusions from the findings. Finally, the higher proportion of missing data in the surgery group (7/21) compared to the conservative group (2/22) may introduce bias and affect the validity of our findings, as differential loss to follow-up can limit comparability and generalizability between groups.

In conclusion, the trends in our data point in the same direction as previous RCTs, showing possible benefits of adrenalectomy on improving blood pressure and metabolic parameters in patients with MACS.

By using defined endpoints and recording antihypertensive medications as DDD, our study addressed several methodological limitations of earlier research. However, the challenges we faced with recruitment and follow-up highlight the difficulties in conducting well-powered RCTs in this patient population, many with significant comorbidities. Continued research with larger cohorts and longer follow-up is essential to refine treatment approaches and optimize outcomes for patients with MACS. The results from the present investigation may be of value for future meta-analyses within this field.

## Supplementary materials



## Declaration of interest

The authors declare that there are no conflicts of interest that could be perceived as prejudicing the impartiality of the research reported.

## Funding

This work did not receive any specific grant from any funding agency in the public, commercial, or not-for-profit sector.

## Data availability

All datasets generated during and/or analyzed during the current study are not publicly available but are available from the corresponding author on reasonable request.
