# Supplementary material for: Randomized trial studying metabolic outcomes and quality of life after adrenalectomy versus conservative management for mild autonomous cortisol secretion
Source: Endocr Connect. 2025 Jul 19;14(7):e250361. doi: 10.1530/EC-25-0361 (PMC12281625; doi:10.1530/EC-25-0361)
Supplement: Supplementary file 1 [file supplementary_materials.pdf]

Supplemental material

Detailed information on assay of hormones

All hormone analyses were performed at the local laboratory at each hospital, and the local reference ranges were used.

-*Haukeland University Hospital*

Serum cortisol was assayed using an in-house developed high performance liquid chromatography tandem mass spectrometry (LCMSMS) (ref). The reference range was 120–600 nmol/L for samples drawn before 10:00 a.m. The assay precision was 4.5–7.4% relative standard deviations (RSD), and the accuracy ranged from 97 to 101% .

ACTH was determined by chemiluminescent immunoassay (Immulite 2000 XPI, Siemens Healthineers, Erlangen, Germany). The lower limit of quantification for ACTH was 1.1 pmol/L. ACTH had a total RSD of 5.0% at 3 pmol/L, and 8.4% at 49 pmol/L.

Urinary cortisol was analysed by LCMS/MS with an analytical precision of RSD 10% at a 140 nmol/L concentration. Free cortisol excretion below 165 nmol/24 h was considered normal.

- *Skåne University Hospital, Lund*

Until September 15th 2015 plasma cortisol was analysed using a 1-step competitive immunoassay (Cobas, Roche Diagnostics). The reference range was 171 to 536 nmol/L, the coefficient of variation was 2.1% at 94.9 nmol/L, and the detection limit was 0.5 nmol/L. After September 15th 2015 plasma cortisol was analysed using a 1-step competitive immunoassay (Cobas Pro, Roche Diagnostics). The reference range was 133 to 537 nmol/L, the coefficient of variation was 2.4% at 117 nmol/L, and the detection limit was 1.5 nmol/L

Plasma ACTH was analysed using a 2-step immunometric sandwich assay (Cobas, Roche Diagnostics), the reference range was 1.6 to 13.9 pmol/L, the coefficient of variation was 5.4% at 1.1 pmol/L, and the detection limit was 0.23 pmol/ L.

Urinary free cortisol was analysed by LCMS/MS. The reference range was 38 to 170 nmol/24 h. Lower quantification limit was 2.0 nmol/L, coefficient of variation was 6.4% at 7.97 nmol/L and 3.5% at 560 nmol/L.

-Gøteborg

Plasma cortisol concentrations were determined by radioimmunoassay (Roche Cobas, Cortisol-II). The coefficient of variation was 7% at 100 nmol/L and 5% at both 570 and 990 nmol/L. Plasma ACTH was measured by electrochemiluminescence assays, with a reference interval of 1.6–14 pmol/L. Urinary free cortisol concentrations were measured by liquid chromatography-tandem mass spectrometry (LC-MS/MS). The system used was an Acquity UPLC with a Xevo TQS, equipped with a BEH C18 1.7 µm 2.1 × 100 mm analytical column, all from Waters.

-Århus

P-Cortisol was determined by electrochemiluminescence immunoassay (Cobas 602, Roche Diagnostics GmbH, Mannheim, Germany). The reference range was 171 – 536 nmol/L for samples drawn between 7:00 and 10:00 a.m. The assay precision was 5,9% relative standard deviations (RSD). The method was traceable to IRMM Institute for Reference Materials and Measurements, Geel, Belgien)/IFCC-451 Panel.

P-ACTH was determined by electrochemiluminescence immunoassay (Cobas 602, Roche Diagnostics GmbH, Mannheim, Germany). The reference range was 1.6 – 13.9 pmol/L and the lower limit of quantification was 0.7 pmol/L. ACTH had a total RSD of 1.3% at 10 pmol/L, and 1.0% at 196 pmol/L. Urinary free cortisol was analyzed by RIA (Radio-Immune-Assay) from DRG (Marburg, Germany). The analytical imprecision was 10.1% at 14.7 nmol/L, 6.7% at 128 nmol/L and 6.2% at 297 nmol/L. Cortisol excretion of 37-300 nmol/24 h was considered normal.

-Imperial

Serum cortisol was measured by a one-step chemiluminescent microparticle immunoassay (Alinity I, Abbott). The reference range for samples collected at 09:00 was 160 – 550 nmol/L. The imprecision (CV%) of the assay, determined by long-term internal quality control (IQC) monitoring, was 4%, 5% and 7%, at 114 nmol/L, 463 nmol/L and 979 nmol/L, respectively. The lower limit of quantitation was 28 nmol/L.

Plasma ACTH was measured by a two-site chemiluminescent immunometric assay (Immulite 2000, Siemens). The reference range for samples collected at 09:00 was <30 ng/L and for samples collected at midnight was < 10 ng/L. The imprecision (CV%) of the assay, determined by long-term IQC monitoring, was 9% and 7% at 25 ng/L and 345 ng/L, respectively. The lower limit of quantitation was 5 ng/L.

Urine free cortisol was measured by a one-step competitive chemiluminescent enzyme immunoassay (Immulite 2000, Siemens) with dichloromethane extraction. The reference range was 50 – 270 nmol/24h. The imprecision (CV%) of the assay, determined by long-term IQC monitoring, was 10%, 6% and 8%, at 64 nmol/L 474 nmol/L and 769 nmol/L, respectively. The lower limit of quantitation was 28 nmol/L.

Supplemental Table 1- showing baseline characteristics and two-year follow-up data of the conservative group.

Conservative treatment group	Baseline<br>n=22	2 y follow-up<br>n=18	P-value
BMI kg/m^2	29.6 (21.2-42.2)	31.2 (21.1-43.1)	0.12
Weight, kg	82 (57-131)	86 (55-126)	0.91
s-creatinine, μmol/L	71(56-156)	69(57-155)	0.59
eGFR, mL/min	72 (37-91)	76 (37-90)	0.33
s-cortisol (morning), nmol/L	517(240-697)	380(227-748)	0.08
p-ACTH morning, pmol/L	1.5 (0.2-3.8)	1.8 (1.1-3.2)	0.86
UFC (24 hour urine cortisol), nmol/24 hour	44 (19-264)	32 (17-76)	0.26
DHEA-S, μmol/L	0.6 (0.17-2.6)	0.47 (0-2.0)	0.55
Cortisol after DST, nmol/L	94 (64-381)	95 (33-231)	0.76
s-glucose baseline, mmol/L	6.0 (3.6-7.1)	5.6 (4.9-8.6)	0.08
s-glucose 120 min., mmol/L	7.5 (5.3-13.2)[a]	7.3 (4.8-18.1)[c]	0.55
HbA1C mmol/mol	39 (32-58)	39 (33-47)	0.21
Total Cholseterol, mmol/L	5.1 (2.1-8.3)	4.7 (2.9-6.2)	0.03
LDL, mmol/L	3.3 (1.2-5.6)	3.0 (1.2-4.5)	0.06
HDL, mmol/L	1.4 (0.9-2.3)	1.4 (0.7-2.4)	0.5
Hypertension, n	18	13	1.0
Diabetes mellitus, n	6	6	0.56
Osteoporosis, n	5	4	1.0
Fractures, n	7	4	0.16

Office syst BP, mmHg[2]	138 (124-186)	138(124-157)	0.65
Office diast BP, mmHg[2]	81(58-112)	81(70-93)	0.78
Mean 24-hour syst BP, mmHg[2]	140(105-185)	130(106-193)	<0.01
Mean 24-hour diast BP, mmHg[2]	82(57-95)	78(63-109)	0.37
Mean day syst BP, mmHg[2]	139(107-182)	130(105-200)	<0.01
Mean day diast BP, mmHg[2]	80(58-99)	78(66-113)	0.54
Mean night syst BP, mmHg[2]	130(98-186)	126(93-168)	0.07
Mean night diast BP, mmHg[2]	69(54-91)	67(53-86)	0.29
Systolic nightly dip, mmHg[2]	11 (-8.7-22)	9 (-14-19)	0.76
Diastolic nightly dip, mmHg[2]	13.5 (-2.5-29.8)	15.7 (-4-24.0)	0.65
Total bone mass back, g/cm^2[2]	1.09 (0.85-1.78)	1.12 (0.76-1.13)	0.22
Total bone mass hip, g/cm^2[2]	0.94 (0.62-1.25)	0.98 (0.65-1.24)	0.14
T-score back[2]	-0.4 (-2.8-4.7)	-0.05 (-3.3-10.0)	0.10
T-score hip[2]	-0.5 (-3.2-2.1)	-0.45 (-2.9-1.8)	0.73
Septum hyperthrophy, N (%)[2]	6 (27.3%)	6 (33.3%)	0.32
Septum thickness, mm	11.5(7.5-15.0)	11.5 (8.0-16.0)	0.37
Daily doses antihypertensive[2]	1.8 (0-5)	2.0 (0-8.5)	0.21
Cholesterol lowering drugs, n	10	10	0.16

Continuous variables are presented as median (range), Categorical variables are given as number (n) and percent (%)

BMI (Body mass index), DST (Dexamethasone suppression test), UFC (24 hour urine free cortisol), BP (Blood pressure), HU (Houndsfield units), DHEA-S (Dehydroepiandrostendione sulfate)

Obese means BMI ≥30 kg/m^2

[1]In non-diabetic patients (n=30)

P-value indicate differences between the conservative and surgery group at baseline

Reference ranges for s-cortisol morning, p-ACTH and UFC are given in supplemental file, as they are slightly different between the hospitals. Reference ranges for DHEA-S (<2.5 μmol/L), cortisol after DST (<50 nmol/L), s-fasting glucose (<7mmol/L), s-glucose 120 min post oral glucose tolerance test (≤11.1 mmol/L).

[2]At baseline, the number of patients with missing data in the conservative was as follows: 24-hour BP (0), OGTT in non-diabetic patients (2), septum thickness (3), and bone density measurement (4). At the two-year follow-up, the following numbers of patients had missing data in the conservative group: office BP (3), DDD (2), 24-hour BP (3), OGTT (4), HbA1c (2), septum thickness (4), and bone density (4).

Supplemental Table 2- showing baseline characteristics and two-year follow-up data of the surgery group.

**Surgery group**	**Baseline** n=21	**2-y follow-up** n=15	**P-value**
BMI kg/m^2	28.0 (21.7-36.0)	28.6 (22.3-34.7)	0.52
Weight,kg	80.7 (62.0-106.8)	77.3 (62.0-206.8)	0.50
s-creatinine, μmol/L	73(48-229)	86(59-118)	<0.01
eGFR, mL/min	79 (19-97)	66 (39-90)	<0.01
s-cortisol (morning)	425 (154-636)	366 (147-504)	0.82
p-ACTH morning,	1.4 (0.2-1.9)	6.5 (3.5-11.1)	<0.01

pmol/L			
UFC (24 hour urine cortisol), nmol/24 hour	64 (2-147)	47 (43-117)	0.59
DHEA-S, µmol/L	1.2 (0.4-4.2)	2.5 (1-4.0)	0.50
Cortisol after DST, nmol/L	101 (55-402)	43 (10-98)	<0.01
p-glucose baseline, mmol/L	5.8 (4.4-8.1)	5.2 (4.4-6.9)	0.4
s-glucose 120 min., mmol/L[2]	7.6 (3.4-15.2)	6.2 (3.8-6.8)	0.25
HbA1C mmol/mol[2]	39 (32-56)	40(35-42)	0.75
Total Cholesterol, mmol/L	5.1 (1.9-7.3)	5.2 (4.6-6.2)	0.49
LDL, mmol/L	3.3 (1.6-5.9)	4.2 (2.7-4.8)	0.59
HDL, mmol/L	1.2 (0.8-3.0)	1.2 (0.9-2.3)	0.93
Hypertension, n	17	10	0.32
Diabetes mellitus, n	7	7	0.32
Osteoporosis, n	4	3	0.16
Fractures, n	4	2	0.56
Office syst BP, mmHg[2]	135(111-180)	125(106-160)	0.20
Office diast BP, mmHg[2]	80(68-97)	75(55-115)	0.17
Mean 24-hour syst BP, mmHg[2]	131 (87-188)	122(107-164)	0.24
Mean 24-hour diast BP, mmHg[2]	79 (59-104)	75 (62-93)	0.33
Mean day syst BP, mmHg[2]	133(96-184)	118(110-160)	0.42
Mean day diast BP, mmHg[2]	83 (66-108)	77 (61-93)	0.43
Mean night syst BP, mmHg[2]	127(76-197)	116(85-177)	0.22

Mean night diast BP, mmHg[2]	71(49-95)	64(48-95)	0.30
Systolic nightly dip, mmHg[2]	9.4(-11.8-20.8)	13.3(-7.1-27.8)	0.43
Diastolic nightly dip, mmHg[2]	13.5(-9.0-26)	11.1(-2.2-36)	0.72
Total bone mass back, g/cm^2	1.07 (0.66-1.31)	1.09 (0.99-1.61)	0.40
Total bone mass hip, g/cm^2	0.94(0.62-1.03)	0.94(0.79-1.22)	0.68
T-score back[2]	-0.9 (-4.3-1.1)	-0.8 (-2.4-2.8)	0.83
T-score hip[2]	-1.2 (-3.0-1.8)	-0.9 (-2.2-1.8)	0.31
Septum hyperthrophy, N (%)[2]	6 (28.6)	3 (20)	1.0
Septum thickness, mm[2]	10.0 (7.2-19.0)	9.6(8.0-12.0)	0.58
Daily doses antihypertensive[2]	1.4 (0-6.5)	2.3(0-6.5)	0.87
Cholesterol lowering drugs, n	8	7	1.0

Continuous variables are presented as median (range), Categorical variables are given as number (n) and percent (%)

BMI (Body mass index), DST (Dexamethasone suppression test), UFC (24 hour urine free cortisol), BP (Blood pressure), HU (Houndsfield units), DHEA-S (Dehydroepiandrostendione sulfate)

Obese means BMI ≥30 kg/m^2

[1]In non-diabetic patients (n=30)

P-value indicate differences between the conservative and surgery group at baseline

Reference ranges for s-cortisol morning, p-ACTH and UFC are given in supplemental file, as they are slightly different between the hospitals. Reference ranges for DHEA-S (<2.5 μmol/L), cortisol after DST (<50 nmol/L), s-fasting glucose (<7mmol/L), s-glucose 120 min post oral glucose tolerance test (≤11.1 mmol/L).

[2]At baseline, the number of patients with missing data in the surgery groups, respectively, was as follows: 24-hour BP (1), septum thickness (5), and bone density measurement (3). At the two-year follow-up, the following numbers of patients had missing data in the surgery group: office BP (8), DDD (3), 24-hour BP (7), OGTT (7), HbA1c (7), septum thickness (10), and bone density (8).

Supplemental Table 3- showing the scores on each subcategory of SF36 in each group.

	**Baseline**		**Follow-up**		**Delta**		
	**Surgery (n=8)**	**Controls (n=15)**	**Surgery (n=8)**	**Controls (n=15)**	**Surgery (n=8)**	**Controls (n=15)**	**P**
**Physical functioning**	78 (71-94)	70 (40-95)	80 (74-100)	68 (48-100)	5 (-2 to 10)	5 (-8 to 8)	0.73
**Role limitations due to physical health**	100 (19-100)	75 (0-100)	75 (13-100)	50 (0-100)	0 (-44 to 0)	0 (-50 to 0)	0.68
**Role limitations due to emotional problems**	100 (100-100)	100 (33-100)	100 (67-100)	67 (0-100)	0 (-25 to 0)	0 (-33 to 0)	0.59
**Energy/Fatigue**	53 (46-74)	40 (25-70)	55 (45-64)	50 (25-70)	-5 (-10 to 10)	5 (-10 to 10)	0.36
**Emotional well-being**	76 (72-87)	76 (52-96)	84 (80-90)	76 (60-92)	8 (-3 to 17)	0 (-4 to 8)	0.27
**Social functioning**	88 (50-100)	75 (50-100)	81 (56-100)	88 (75-100)	-6 (-13 to 9)	0 (-13 to 25)	0.51
**Pain**	51 (38-82)	68 (45-90)	58 (28-98)	58 (33-90)	0 (-9 to 12)	0 (-13 to 10)	0.37
**General health**	48 (36-69)	65 (35-85)	53 (41-69)	60 (30-85)	5 (-10 to 15)	-5 (-10 to 0)	0.29

Data is presented as median (25th to 75th percentile).
